# Research on the model of staggered tooth phase to reduce vibration of single-stage gear transmission system: Theoretical analysis and experiments

**DOI:** 10.1371/journal.pone.0297936

**Published:** 2024-04-05

**Authors:** Shaoshuai Hou, Bin Li, Derong Zhu, Haichao Ye, Chunpeng Zhang

**Affiliations:** 1 School of Intelligent Manufacturing, Luoyang Institute of Science and Technology, Luoyang, Henan, China; 2 State Key Laboratory of Mechanical Transmissions, Chongqing University, Chongqing, China; 3 School of Machinery and Automation, Weifang University, Weifang, Shandong, China; The University of British Columbia, AUSTRALIA

## Abstract

Aiming at the problems of high vibration and high noise in gear transmission systems, a model of gear with staggered tooth phase structure(GSTPS) for reducing vibration is proposed. Without changing the overall structure of the gear transmission system, the purpose of reducing mesh stiffness fluctuations is achieved by staggering adjacent gears at a certain angle along the axis, thereby the vibration of the gear transmission system could be reduced. The characterization method of time-varying mesh stiffness of the GSTPS is studied. Then, the impact of different staggered tooth phases(STP) on reducing vibration of the transmission system are researched, and the basis for selecting the optimal STP are obtained. The experimental platform for reducing vibration with STP is established. And some experimental studies were conducted to validate the theoretical model.

## Introduction

Gear transmission is an important form of power transmission which is widely used in the fields of aerospace, naval ships, automotive, and other fields. With the increasing requirements of national defense construction, gear transmission is developing towards the direction of high speed and high load [1]. This development trend often leads to greater vibration and noise. Furtherly, high vibration may cause premature fatigue damage to gears and bearings, and even lead to serious consequences, resulting in reduced overall reliability and reduced service life [2]. The mesh stiffness is one of the main excitation causing vibration and noise in the gear dynamic research [3]. Therefore, it has important research significance that reducing the fluctuation of mesh stiffness, and the vibration of the gear transmission system.

Many scholars have carried out a lot of research on the method of calculating mesh stiffness and reducing mesh stiffness fluctuations. Chen [4] established an analytical model for time-varying mesh stiffness of spur gears which considered the deflection of tooth and fillet-foundation. Xie [5] established a model for calculating mesh stiffness of spur gears that takes into account the structure coupling effect. The results show that the structure coupling effect significantly reduces the overall meshing stiffness value in the double tooth meshing region. Considering the effect of structure coupling and axial deflection, Hou [6] researched the calculating method of mesh stiffness of helical gear, and then, pointed out the principles for selecting the basic parameters of gears with minimum mesh stiffness fluctuations. By bridging over the gear teeth with external stiffness elements, Andary [7] introduces a new potential energy-based design method to calculate mesh stiffness. Raghuwanshi [8, 9] measured the mesh stiffness of spur gear based on experimental modal analysis and digital image technology, respectively. Based on finite element theory and load contact analysis, Chen and Huangfu [10] established a model for calculating mesh stiffness considering complex fillet-foundation structures. The results show that a reduction in the thickness of rim and web would result in a reduction in mesh stiffness. Yu [11] proposed a screw analysis method of SNCG compound transmission based on the gear meshing theory, screw theory and calculus. Chen and Shao [12] have studied the influence of tooth profile modification on mesh stiffness, and the results showed that by appropriately selecting profile modification parameters, the mesh stiffness could be optimized to obtain the minimum fluctuation. Wang [13] has studied the effects of profile modification, crown modification and helical angle modification on mesh stiffness respectively. Deng [14] has studied the effect of profile modification on mesh stiffness and transmission performance of asymmetric gears. Hu [15] has studied the influence of profile modification on mesh stiffness and further obtained the dynamic response of the transmission system. The results show that the influence of long tooth profile modification on mesh stiffness fluctuation in the double tooth meshing region is more obvious. Liu [16] studied the effect of profile modification on the vibration response of a single stage gear transmission system. The results show that for different modification lengths and patterns, the distribution of dynamic load coefficients follows a "V" pattern as the amount of modification increases. Motahar [17] studied the influence of profile modification on the vibration response of bevel gears, and the results showed that through increasing tooth profile modification, the average value of the dynamic responses would be decreased intensely for both dynamically and statically optimized gear pairs.

Not only are tooth surface modifications used to reduce vibration by many scholars, but there are also many new methods to reduce gear vibration. Kong [18] has studied the dynamic characteristics of gear systems with thin-walled structures. Geng [19] has proposed a rigid-flexible coupling gear structure that includes rings, metal rubber, and wheel hubs, then compared the vibration characteristics with pure rigid gear pairs, and explained the advantages of rigid-flexible coupling gear in reducing vibration. Lai [20] has proposed a rigid-flexible coupling dynamic model for the floating ring, and revealed the vibration mechanism of the floating ring. Zhu [21] has studied the dynamic characteristics of planetary gear system with flexible pin structures, and the results show that reducing stiffness of flexible pins could reduce the number of low-frequency natural frequency components, which is beneficial to reducing resonance. He [22] has studied the vibration characteristics of planetary gear trains with floating sun gear under different operating conditions. Mo [23] has studied the dynamic characteristics of a herringbone planetary gear train with flexible support and floating sun gear structure, and the results show that floating sun gear is beneficial to load distribution and transmission system stability. Wang [24] has studied the influence of the position and number of bearing supports on the load sharing characteristics of planetary gear trains. Xiao [25] applied particle damping technology to gear transmission systems, and both theory and experiment have verified that this method has certain vibration reduction performance. Firrone [26] has proposed a passive vibration reduction method based on an annular damper, which reduces the vibration amplitude by optimizing the material and geometric properties of the annular damper. Yang [27] has optimized the parameters of the weight reduction holes in the gear blank from the perspective of passive vibration reduction, and improved the vibration behavior of lightweight gears. Considered factors such as gear meshing deformation and thermal effects, Xu [28] has studied the mesh stiffness of plastic gear pairs, and achieved the purpose of reducing fluctuation of mesh stiffness by changing the material of the gear. Sim [29] has used hybrid gears made of fiber reinforced polymer composites and steel for automotive gearboxes. Compared to 100% steel gears, the vibration acceleration of hybrid gears is reduced.

In fact, the method of tooth surface modification is highly dependent on load, and the modification parameters under one load are difficult to adapt to another load condition. The floating support structure is relatively complex in processing and assembly processes, and the cost of using new materials for gear vibration reduction is high. This paper proposes a model of GSTPS to reduce vibration, which achieves the effect of reducing mesh stiffness fluctuations without changing the overall structure of the gear transmission system, thereby reducing the vibration of the gear transmission system.

The outline of this article is as follows: the literature on vibration reduction methods for gear transmission systems are summarized and the existing problems are pointed out in section 1. Then, the GSTPS is described, the calculating method of mesh stiffness of GSTPS is derived, the dynamic model of the transmission system based on a pair of gear in section 2. The experimental platform for vibration reduction with STP is built in section 3. The influence of different STP on vibration responses are studied in section 4. The conclusion of this research are given in section 5.

## The model of STP to reduce vibration

### Description of the GSTPS

**Fig 1:**
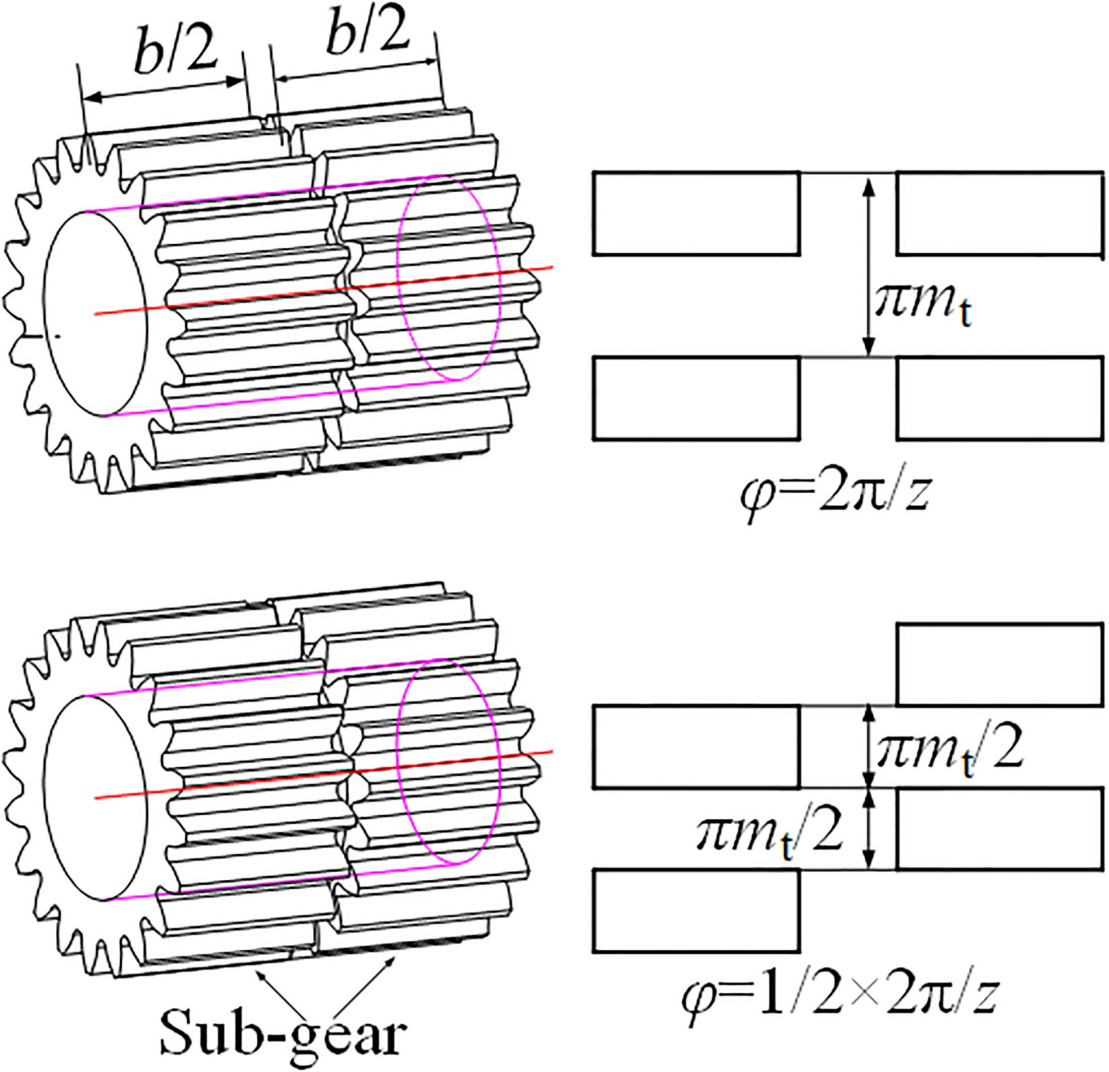
The gear with staggered tooth phase structure.

For a traditional gear, there is no meshing phase difference. However, if a gear with width *b* is divided into two gears with width *b*/2, and the sub-gears are staggered by a certain angle along the rotating axis direction, there could be meshing phase differences. This change does not affect the gear transmission ratio, satisfies the gear meshing principle, and achieves mesh stiffness complementarity through overall meshing. In this way, the meshing phase difference could be adjusted to improve the gear transmission performance. This method is called as the method of staggered tooth phase to reduce vibration. The corresponding angle is called as the staggered tooth phase angle(STPA), as shown in Fig 1.

For single stage transmission system containing two meshing pairs, the relationship between the STP and the STPA is defined as,

$$p=\frac{\varphi }{2\pi /z}$$ (1)

Where, *p* denotes STP, *z* denotes tooth number of driving gear, *φ* denotes STPA. Due to the periodicity of gear engagement, the STPA could not exceed a standard pitch angle 2π/*z*. The relationship between STPA and the angle at which one side gear rotates *φ*_0_ is

$$\varphi =\text{mod}\left(\varphi_{0},\frac{2\pi }{z}\right)$$ (2)

Where, mod(*x*, *y*) denote mod function, namely, mod(*x*, *y*) equal to the remainder value of *x* divided by *y*.

With the reason that there exists a certain angle around the rotating axis between the sub-gears, the corresponding teeth on the two sub-gears would not enter the actual meshing region simultaneously. This results in different positions of the two sub-gears in the multi-teeth meshing region or few-teeth meshing region at the same time.

**Fig 2:**
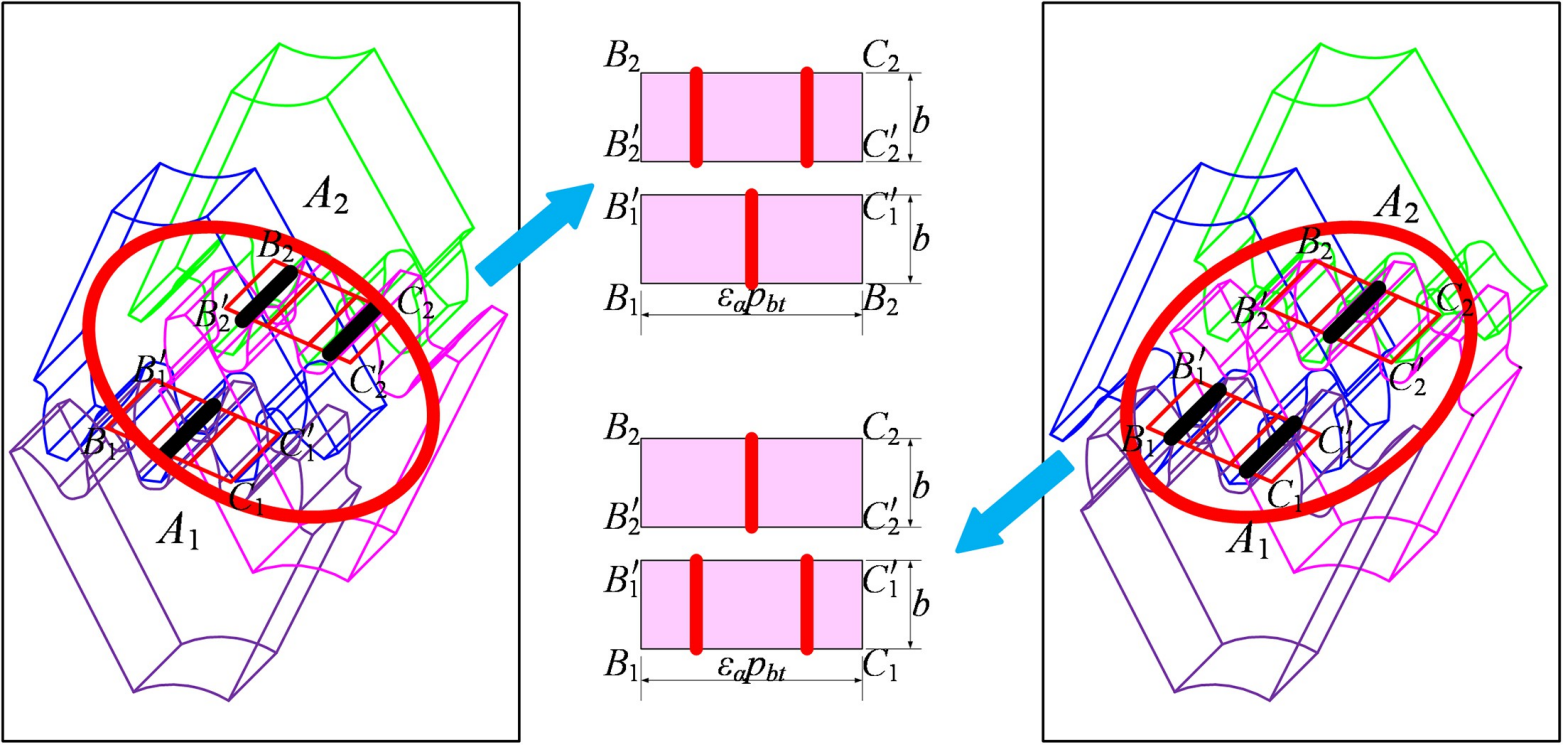
The meshing principle of the staggered phase gears.

For standard spur gears, contact ratio is 1<ε<2. The meshing process always alternates periodically between single-tooth and double-tooth meshing region. But for the GSTPS, when one meshing gear pair is in the single tooth meshing region, the other meshing gear pair would be in the double teeth meshing region through adjusting the STPA, and vice versa. Therefore, the two meshing pairs with STPA could be understood as two meshing pairs with different initial meshing positions, as shown in Fig 2.

### The mesh stiffness of a certain spur gear pair

**Fig 3:**
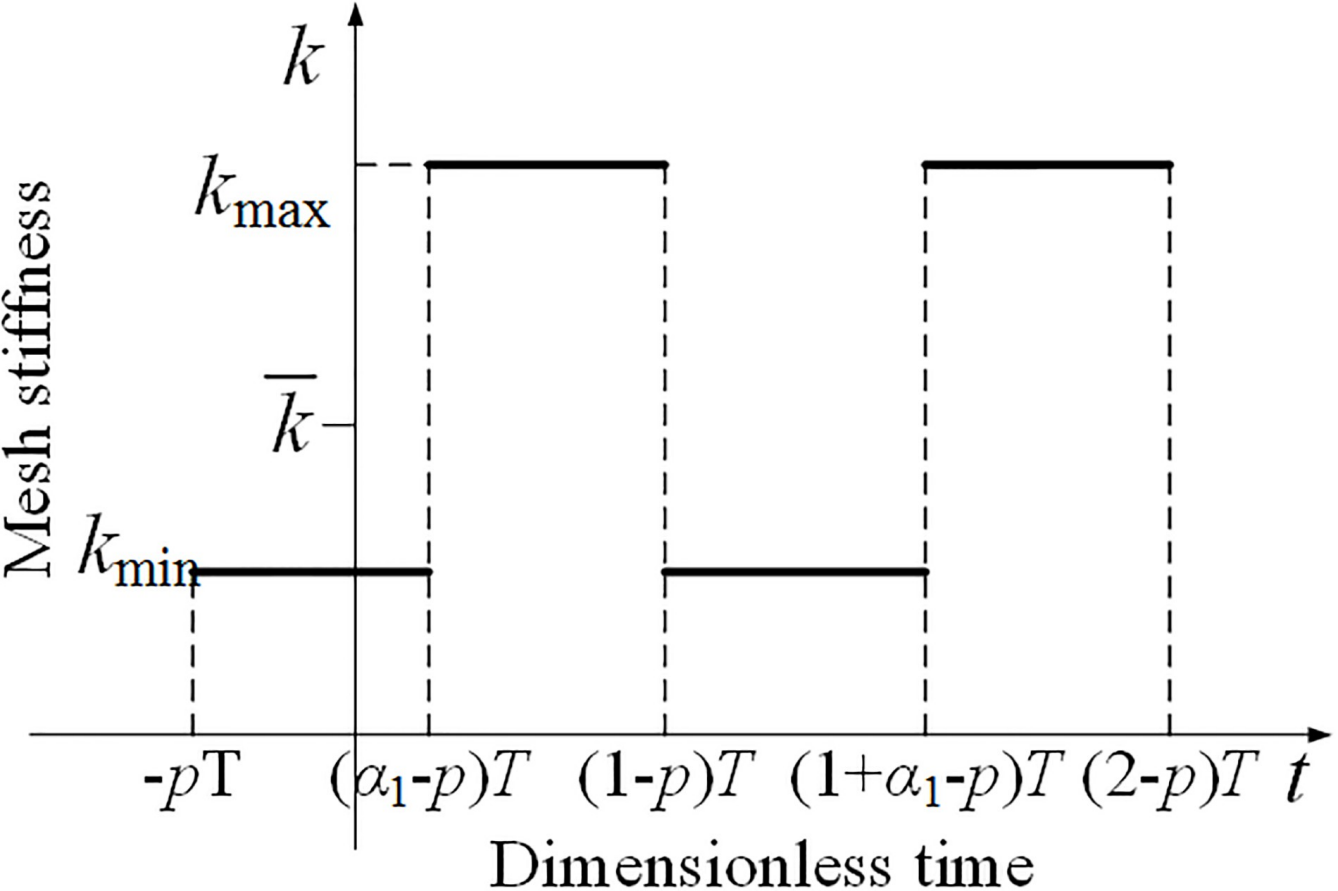
The mesh stiffness curve of spur gear at any meshing phase.

The mesh stiffness curve of spur gear could be written as a rectangular wave [30], as shown in Fig 3.

Therefore, the mathematical expression for time-varying mesh stiffness of spur gear pairs within any meshing cycle could be written as,

$$k\left(t\right)=\left\{\begin{array}{l} k_{\text{min}}\;\;\;\;\;nT<t<\left(n+\alpha_{1}-p_{0}\right)T\\ k_{\text{max}}\;\;\;\;\left(n+\alpha_{1}-p_{0}\right)T<t<\left(n+1-p_{0}\right)T\\ k_{\text{min}}\;\;\;\;\left(n+1-p_{0}\right)<t<\left(n+1\right)T \end{array}\right.\;\;\;\;n=0,1,2\cdots$$ (3)

Where, *k*_max_, *k*_min_ denote the maximum value and minimum value of mesh stiffness of spur gear respectively. *T* is meshing period, and *p*_0_ is the phase when tooth entering the meshing region. Obviously, for GSTPS, *p*_1_≠*p*_2_. *α*_1_ is a coefficient related to contact ratio.

$$\alpha_{1}=1-\varepsilon_{\alpha }^{\prime }$$ (4)

Where, $\varepsilon_{\alpha }^{\prime }=\text{mod}\left(\varepsilon_{\alpha },1\right)$, *ε*_α_ denote contact ratio.

To simplify the calculation, the mesh stiffness is written as the sum of the average value and the varying value,

$$k\left(t\right)=\bar{k}+\Delta k\left(t\right)$$ (5)

Where, $\bar{k}$ denotes the average value of mesh stiffness within one meshing cycle, Δ*k*(*t*) is the varying value of mesh stiffness which also exhibits periodic changes over time.

According to the geometric relationship of the rectangular wave of the mesh stiffness curve, the average value of the mesh stiffness within a meshing cycle could be derived as,

$$\bar{k}=k_{\text{min}}\alpha_{1}+k_{\text{max}}\left(1-\alpha_{1}\right)$$ (6)

In order to study the fluctuation law of time-varying mesh stiffness, the varying value of mesh stiffness is written as Fourier series,

$$\Delta k\left(t\right)=\sum_{n=1}^{\infty }\left(a_{n}\text{cos}\frac{2n\pi t}{T}+b_{n}\text{sin}\frac{2n\pi t}{T}\right)$$ (7)

Where,

$$\left\{\begin{array}{l} a_{n}=\frac{2\left(k_{\text{min}}-k_{\text{max}}\right)}{n\pi }\text{sin}\left(n\pi \alpha_{1}\right)\text{cos}\left[n\pi \left(\alpha_{1}-2p\right)\right]\\ b_{n}=\frac{2\left(k_{\text{min}}-k_{\text{max}}\right)}{n\pi }\text{sin}\left(n\pi \alpha_{1}\right)\text{sin}\left[n\pi \left(\alpha_{1}-2p\right)\right] \end{array}\right.$$ (8)

### The mesh stiffness of GSTPS

For the GSTPS, the mesh stiffness of the adjacent gear pairs could be assumed as *k*^(1)^(*t*) and *k*^(2)^(*t*), respectively. The superscripts (1) and (2) are used to represent the two gear pairs, respectively. And the initial meshing phases of the adjacent gear pairs are defined as *p*^(1)^ and *p*^(2)^. Therefore, the mesh stiffness of GSTPS could be written as,

$$\begin{array}{l} k^{\left(1\right)}\left(t\right)=\bar{k}+\Delta k^{p\left(1\right)}\left(t\right)\\ k^{\left(2\right)}\left(t\right)=\bar{k}+\Delta k^{p\left(2\right)}\left(t\right) \end{array}$$ (9)

Where, $\bar{k}$ denotes the average value of the mesh stiffness of a meshing pair within one meshing cycle. Δ*k*^*p*(1)^ and Δ*k*^*p*(2)^ denote the varying value of mesh stiffness, as for *p*^(1)^≠*p*^(2)^, obviously, Δ*k*^*p*(1)^≠Δ*k*^*p*(2)^.

With the reason that the GSTPS could be seen as two gear pairs participate in meshing simultaneously, the mesh stiffness of the single side meshing pair of the GSTPS *k*_*d*_(*t*) could be obtained by averaging the mesh stiffness of the two meshing pairs. That is to say, it could be calculated as,

$$k_{d}\left(t\right)=\frac{1}{2}\left[k^{\left(1\right)}\left(t\right)+k^{\left(2\right)}\left(t\right)\right]$$ (10)

After further organization,

$$k_{d}\left(t\right)=\bar{k}+\frac{1}{2}\left[\underset{\Delta k}{\underset{︸}{\Delta k^{p\left(1\right)}\left(t\right)+\Delta k^{p\left(2\right)}\left(t\right)}}\right]$$ (11)

Where, *Δk* denotes the varying value of the mesh stiffness of the GSTPS.

By writing the varying value in form of Fourier series, the expression could be obtained as,

$$\begin{array}{l} \Delta k=\sum_{n=1}^{\infty }\left\{a_{n}^{p\left(1\right)}\text{cos}\frac{2n\pi t}{T}+b_{n}^{p\left(1\right)}\text{sin}\frac{2n\pi t}{T}\right\}+\sum_{n=1}^{\infty }\left\{a_{n}^{p\left(2\right)}\text{cos}\frac{2n\pi t}{T}{+}_{n}^{p\left(2\right)}\text{sin}\frac{2n\pi t}{T}\right\}\\ \;\;\;\;\;\;=\sum_{n=1}^{\infty }\left\{\left[\underset{a_{n}^{d}}{\underset{︸}{a_{n}^{p\left(1\right)}+a_{n}^{p\left(2\right)}}}\right]\text{cos}\frac{2n\pi t}{T}+\left[\underset{b_{n}^{d}}{\underset{︸}{b_{n}^{p\left(1\right)}+b_{n}^{p\left(2\right)}}}\right]\text{sin}\frac{2n\pi t}{T}\right\}\\ \;\;\;\;\;\;=\sum_{n=1}^{\infty }\left\{\sqrt{{\left(a_{n}^{d}\right)}^{2}+{\left(b_{n}^{d}\right)}^{2}}\text{sin}\left(\frac{2n\pi t}{T}+\varphi \right)\right\} \end{array}$$ (12)

Where, $\sqrt{{\left(a_{n}^{d}\right)}^{2}+{\left(b_{n}^{d}\right)}^{2}}$ is initial meshing phase of GSTPS. And the $a_{n}^{d}$, $b_{n}^{d}$ denote the coefficient of the fourier series. For the convenience of calculation, *J* is used to represent the *n*^th^ order amplitude,

$$J=\sqrt{{\left(a_{n}^{d}\right)}^{2}+{\left(b_{n}^{d}\right)}^{2}}$$ (13)

So, it could be obtained as,

$$\Delta k=\sum_{n=1}^{\infty }\left\{J\cdot \text{sin}\left(\frac{2n\pi t}{T}+\varphi \right)\right\}$$ (14)

Where, T denotes the mesh cycle, *φ* denotes meshing initial phase of the GSTPS.

For GSTPS, *p*^(1)^≠*p*^(2)^, it could be supposed that *p*^(1)^ = 0 and *p*^(2)^ = *p*. Therefore, the expressions of *an d* and *bn d* could be derived as,

$$\left\{\begin{array}{l} a_{n}^{d}=a_{n}^{p\left(1\right)}+a_{n}^{p\left(2\right)}=\frac{2\left(k_{\text{min}}-k_{\text{max}}\right)}{n\pi }\text{sin}\left(n\pi \alpha_{1}\right)\left\{\text{cos}\left(n\pi \alpha_{1}\right)+\text{cos}\left[n\pi \left(\alpha_{1}-2p\right)\right]\right\}\\ b_{n}^{d}=b_{n}^{p\left(1\right)}+b_{n}^{p\left(2\right)}=\frac{2\left(k_{\text{min}}-k_{\text{max}}\right)}{n\pi }\text{sin}\left(n\pi \alpha_{1}\right)\left\{\text{sin}\left(n\pi \alpha_{1}\right)+\text{sin}\left[n\pi \left(\alpha_{1}-2p\right)\right]\right\} \end{array}\right.$$ (15)

Supposed that,

$$Q_{z}=\frac{2\left(k_{\text{min}}-k_{\text{max}}\right)}{n\pi }\text{sin}\left(n\pi \alpha_{1}\right)$$ (16)

It could be derived that,

$$\left\{\begin{array}{l} a_{n}^{d}=Q_{z}\left\{\text{cos}\left(n\pi \alpha_{1}\right)+\text{cos}\left[n\pi \left(\alpha_{1}-2p\right)\right]\right\}\\ b_{n}^{d}=Q_{z}\left\{\text{sin}\left(n\pi \alpha_{1}\right)+\text{sin}\left[n\pi \left(\alpha_{1}-2p\right)\right]\right\} \end{array}\right.$$ (17)

So, the *n*^th^ order amplitude of the mesh stiffness of the GSTPS could be obtained,

$$\begin{array}{l} J=\sqrt{{\left(a_{n}^{d}\right)}^{2}+{\left(b_{n}^{d}\right)}^{2}}\\ =\left|Q_{z}\right|\sqrt{{\left\{\text{cos}\left(n\pi \alpha_{1}\right)+\text{cos}\left[n\pi \left(\alpha_{1}-2p\right)\right]\right\}}^{2}+{\left\{\text{sin}\left(n\pi \alpha_{1}\right)+\text{sin}\left[n\pi \left(\alpha_{1}-2p\right)\right]\right\}}^{2}}\\ =\left|\sqrt{2}Q_{z}\text{cos}\left(n\pi p\right)\right| \end{array}$$ (18)

It could be found that the mesh stiffness of the GSTPS is not only related to the contact ratio, but also to the STP.

**Fig 4:**
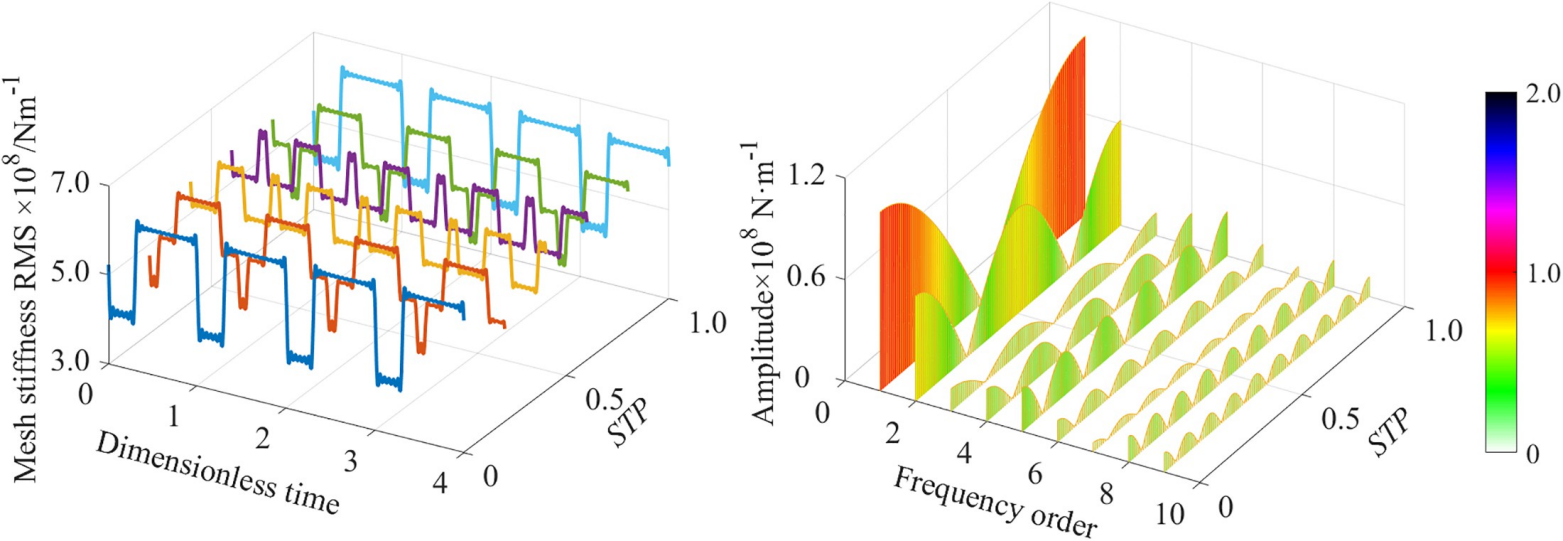
The mesh stiffness of the spur gear with STP. (a) Time domain diagram. (b) Frequency domain diagram.

Taking a set of the spur gear parameters as an example, the time-domain and frequency-domain curves of the mesh stiffness of a pair of spur gears at different staggered tooth phase angle(STPA) are presented, as shown in Fig 4.

It can be observed that for the meshing stiffness of the GSTPS, when the STP *p* = 0 (no GSTPS is generated) or *p* = 1 (the STP is equal to one tooth pitch angle or an integer multiple of one tooth pitch angle), the amplitude of the mesh stiffness curve on the time domain graph reaches its maximum value. When the STP *p*≠0 and *p*≠1, the amplitude of the mesh stiffness is smaller, that is to say, the amplitude of the mesh stiffness fluctuation of the GSTPS is inevitably smaller than that of the ordinary gear pair. In the frequency domain diagram, the different STP results in different amplitudes of the mesh stiffness corresponding to the same order, that is to say, the amplitude of the nth harmonic shows a wave-like variation with the variation of the STP. The minimum value is taken at (2*i*-1)/(2*n*) (where *i* = 1,2…*n*), and the maximum value is taken at *i*/*n* (where *i* = 0,1,2…*n*).

### The dynamic equation of transmission system

**Fig 5:**
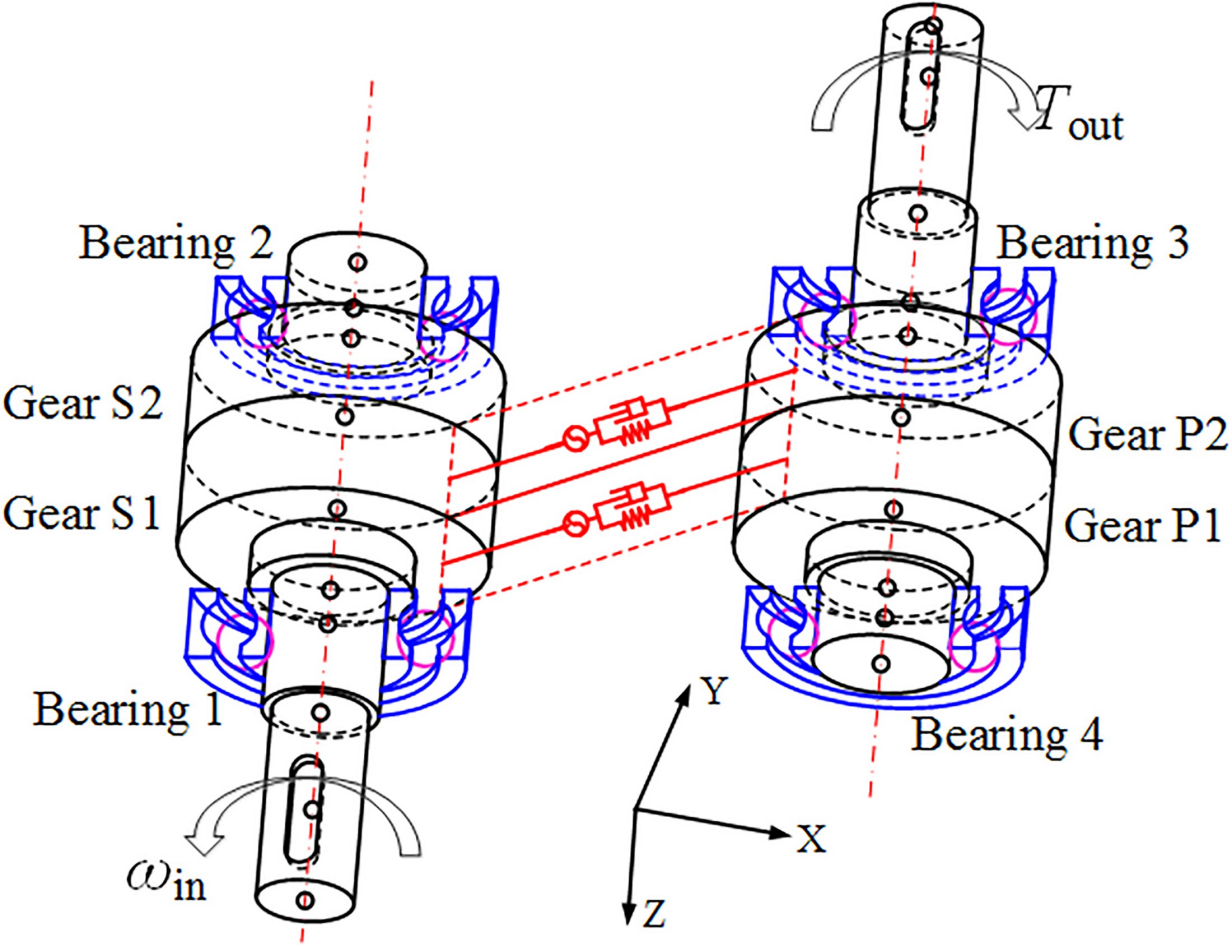
The dynamic model of a single stage spur gear transmission system.

In order to study the effect of the STP on the vibration reduction of gear transmission systems, a dynamic model of parallel-stage spur gear transmission systems was established. The diagram of the transmission system is shown in Fig 5.

The transmission shafts are divided into different shaft segments based on key nodes such as the diameter changes, the position of power input/output, gear meshing and bearing supporting, and so on. Assuming that the two nodes of the shaft segment element are subjected to bending, torsion, and axial forces respectively, their stress states could be analyzed by using the theory of spatial beam elements. The classic basic beam elements commonly used in practical engineering are based on the Kirchhoff assumption, which means that the normal of the mid plane of the beam element coincides before and after deformation. In other word, the shear stress in the cross-section of the beam element is assumed equal to zero. Due to the shear deformation is neglected by Euler-Bernoulli beam elements, it is only suitable for the situations where the ratio of length to diameter of the beam is relatively large. In actual mechanical system, there are many shaft segments with small ratio of length to diameter, and the shear deformation could not be ignored. Timoshenko beam elements take into account shear deformation. Therefore, Timoshenko beam elements is employed to establish the dynamic model of shaft segment. Based on the equivalence principles of frequency approximation and moment of inertia approximation, each shaft segment is equivalent to a two nodes Timoshenko beam element. The dynamic equation of the two node Timoshenko beam element system could be expressed as,

$$M_{ij}{\ddot{X}}_{ij}+\left(C_{ij}+\Omega G_{ij}\right){\dot{X}}_{ij}+K_{ij}X_{ij}=0$$ (19)

Where, ***M***_*ij*_, ***K***_*ij*_, ***G***_*ij*_, ***C***_*ij*_ denote mass matrices, stiffness matrices, gyroscope matrices, and damping matrices of the Timoshenko beam element, respectively. ***X*** denotes displacement of the two nodes of shaft segments. *Ω* denotes rotating speed. The specific expression can be found in reference [31].

The meshing relationship of GSTPS could be equivalent to the connection of two base circular cylinders through a spring-damping element. Therefore, the dynamic differential equation of the meshing element could be written as

$${\text{M}}_{sp}{\ddot{q}}_{sp}+{\text{C}}_{sp}{\dot{q}}_{sp}+{\text{K}}_{sp}{\text{q}}_{sp}={\text{F}}_{spe}$$ (20)

Where, ***M***_*sp*_, ***K***_*sp*_, ***C***_*sp*_ denote mass matrices, stiffness matrices, and damping matrices of the meshing element, respectively. ***q***_*sp*_ represents the displacement of the meshing node. ***F***_*spe*_ represents external load excitation such as meshing error.

The bearing is equivalent to a spring-damping element, where the bearing node on the transmission shaft is directly connected to the gearbox through spring-stiffness and damping. The dynamic equation of the connection element could be written as,

$${\text{M}}_{\text{bi}}{\ddot{X}}_{\text{bi}}{\text{+C}}_{\text{bi}}{\dot{X}}_{\text{bi}}{\text{+K}}_{\text{bi}}{\text{X}}_{\text{bi}}=\text{0}$$ (21)

Where, ***X***_*bi*_ represents the displacement column vector of bearing node. ***M***_*bi*_, ***C***_*bi*_ and ***K***_*bi*_ represent the mass matrix, damping matrix and stiffness matrix of bearing node respectively.

According to the geometric structure of the transmission system, a dynamic model of the transmission system of GSTPS is established, which considering the time-varying mesh stiffness and meshing error. The mass, stiffness, damping, gyroscopic matrix and external load column vector of each element are assembled according to the coordinate displacement of the nodes, so the overall dynamic equation of the transmission system of GSTPS could be written as,

$${\text{M}}_{\text{z}}{\ddot{X}}_{\text{z}}{+\text{C}}_{\text{z}}{\dot{X}}_{\text{z}}{+\text{K}}_{\text{z}}{\text{X}}_{\text{z}}={\text{F}}_{\text{z}}+{\text{F}}_{\text{e}}$$ (22)

Where, ***M***_**z**_, ***K***_**z**_, ***C***_**z**_, ***G***_**z**_ denote the comprehensive mass matrix, stiffness matrix, damping matrix and gyro matrix of the transmission system, ***F***_***z***_ is the external load vector, and ***F***_***e***_ is the error excitation vector.

## Experimental testing

### Implementation plan of GSTPS

**Fig 6:**
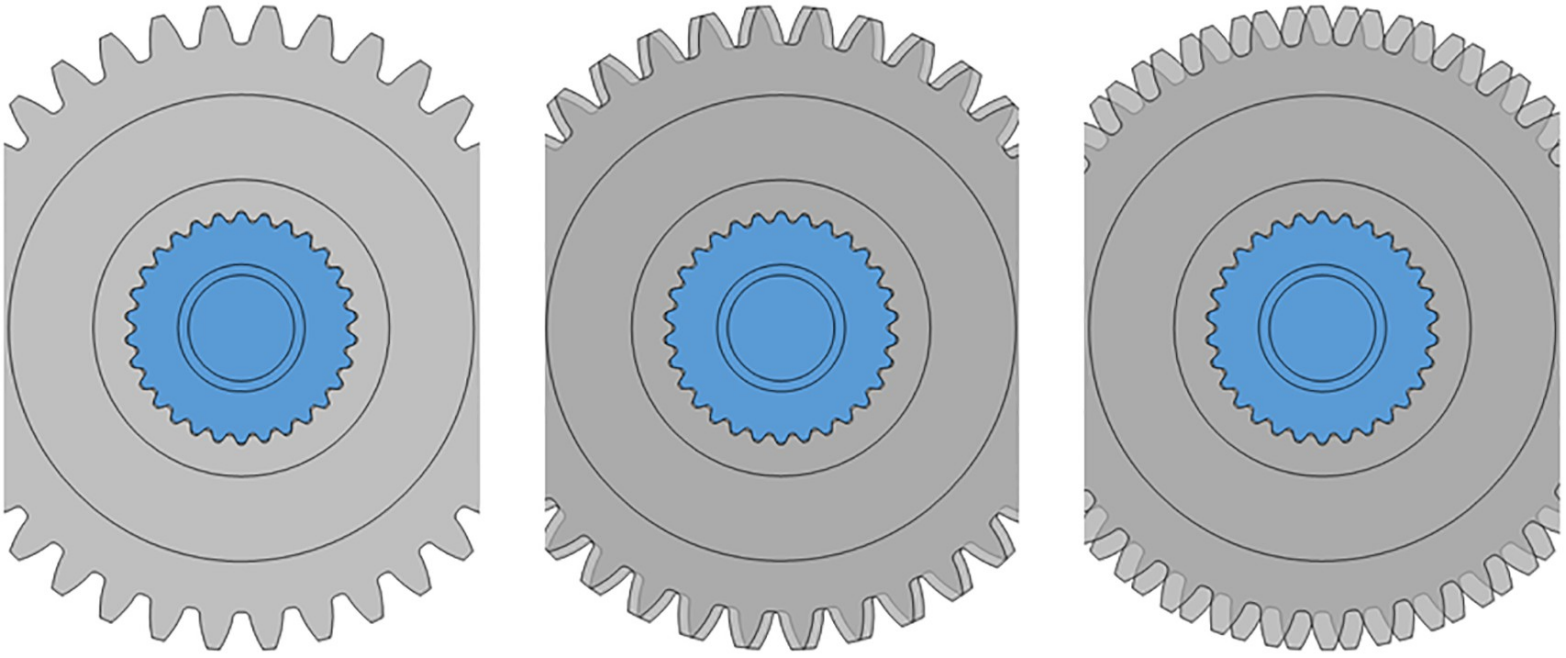
The implementation principle of the gear with different staggered tooth phase.

To verify the theoretical model of the GSTPS and reduce the errors introduced by the processing and assembly of experimental parts, a spline structure is machined on the inner hole of the experimental gear. The reason is for guaranting the accuracy of rotation displacement around the axis. The number of spline teeth *z*_*s*_ is determined jointly by the number of gear teeth *z*_*p*_ and the STP. For two adjacent gears, when the gears are staggered by 0 spline teeth, the STP equal to zero, as shown in Fig 6(a). When the gears are staggered by one spline tooth, that is to say, the second gear rotates 2π/*z*_*s*_ degrees relative to the first gear, the STPA *φ*_1_ = mod(2π/*z*_*s*_, 2π/*z*_*p*_), and the STP *p*_1_ = φ_1_/(2π/ *z*_*p*_), correspondingly, as shown in Fig 6(b). When the gears are staggered by two spline teeth, as shown in Fig 6(c), the second gear rotates 2π/*z*_*s*_×2 degrees relative to the first gear, the STPA *φ*_2_ = mod(2π/*z*_*s*_×2, 2π/*z*_*p*_), and the STP *p*_2_ = φ_2_/(2π/*z*_*p*_), correspondingly. Therefore, different STA could be determined based on the different number of the staggered spline teeth.

### Test bench of GSTPS

**Fig 7:**
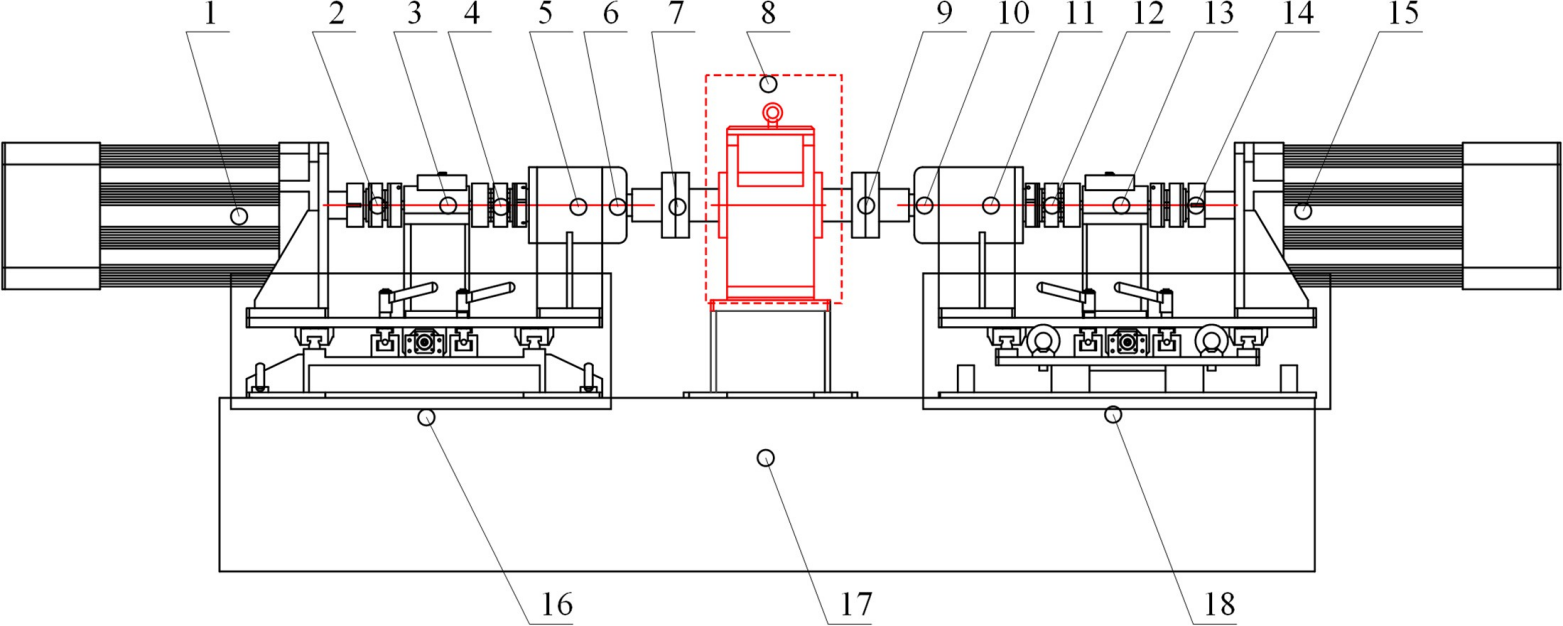
The schematic diagram of the staggered tooth phase experimental platform.

**Fig 8:**
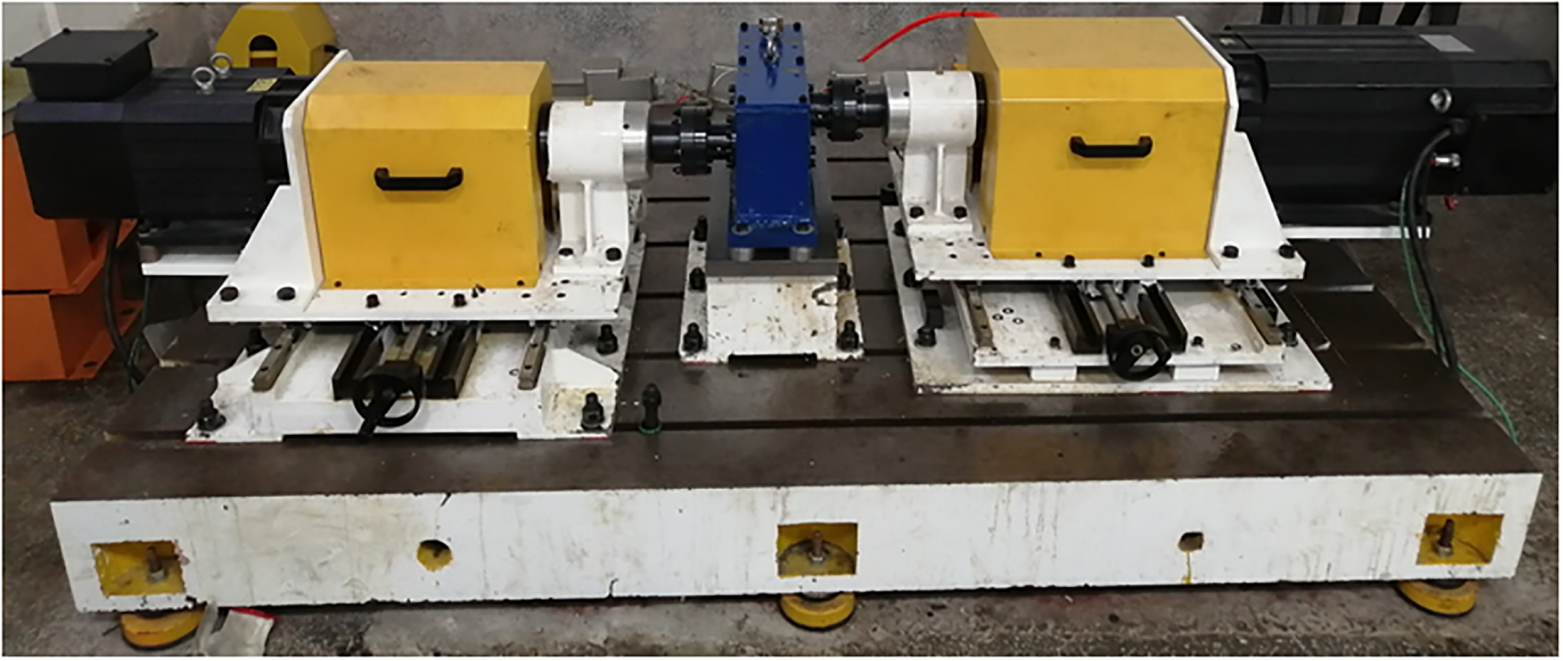
The sketch of the test bench of GSTPS.

The working principle of the experimental platform is shown in Fig 7, and the overall structure of the experimental platform is shown in Fig 8. The main components are as follows: 1-drive motor; 2/4/7/9/12/14-Couplings; 3/13-Torque speed sensor; 5/11- Bearing seat; 6/10- Grating sensor; 8- Experimental gearbox; 15- Load motor; 16/17/18- Experimental bench.

**Fig 9:**
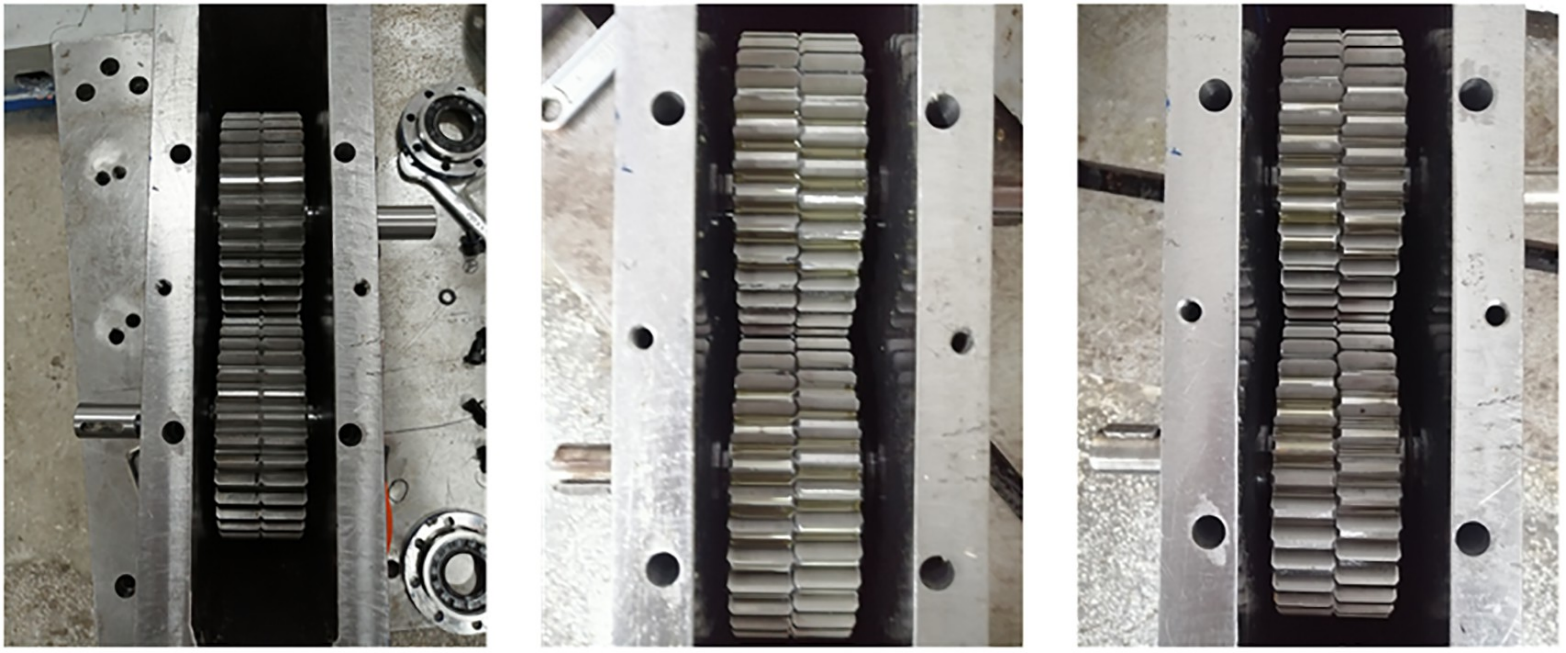
The installation diagram of gears with different staggered tooth phases.

According to the meshing principle of the GSTPS, the gears with different STP are installed, as shown in Fig 9. And the bath oil lubrication is adopted.

### Sensor layout

**Fig 10:**
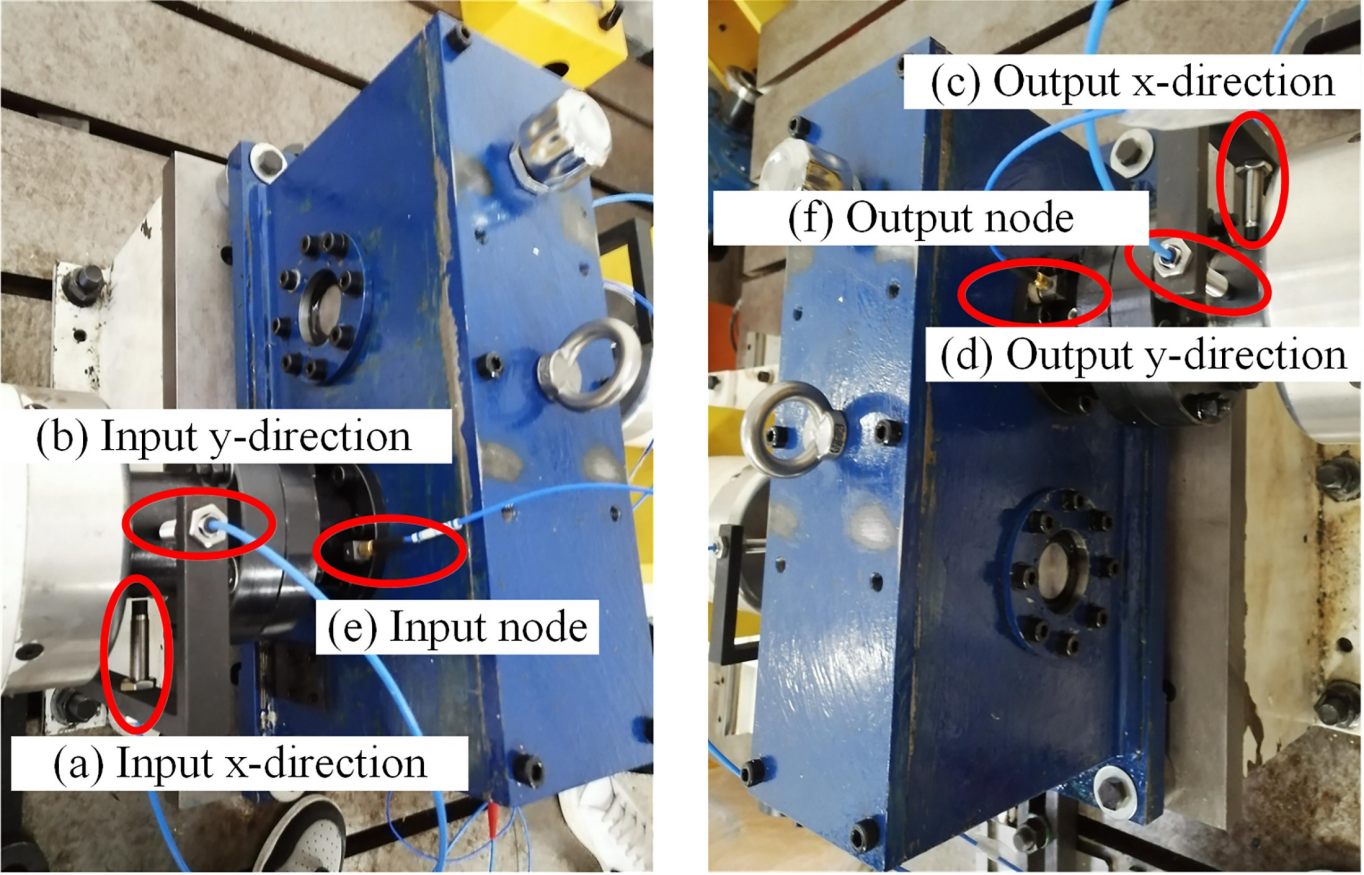
The sketch of the sensor layout.

The vibration displacement sensor and acceleration sensor are detachable sensors. Each probe of the displacement sensor can only test one direction. In order to accurately obtain the radial vibration parameters of the transmission shaft, it is necessary to fix the displacement sensor on the experimental platform through a z-shaped bracket in two perpendicular directions. The measurement points for vibration displacement are arranged at points a/b/c/d. The vibration acceleration sensor could test the vibration response in three directions, so only one sensor needs to be arranged at the input and output points, respectively. And the measurement points for vibration acceleration are arranged at e/f points, as shown in Fig 10.

The parameters of the sensor are shown in Table 1.

**Table 1:** The basic parameters of sensors.

	Type	Sensitivity	Range
Signal acquisition card	NI 9234	8.0 mV/μm	2mm
Displacement sensor	WT0182-A50-B00-C00	8.0 mV/μm	±10V
Acceleration sensor	CT1002LC	20mv/g	±5V

## Results and discussion

### The basic parameters

In this study, a single stage spur gear transmission system was considered, and the basic parameters of the gears are shown in Table 2. The bearing support stiffness was extracted from the Romax software, as shown in Table 3.

**Table 2:** The basic parameters of the gear.

	Modulus *m*/mm	Tooth *z*_1_/*z*_2_	Pressure angle *α*/°	Width *b*/mm	Contact ratio ε
Value	4	36/36	20	26/26	1.692

**Table 3:** The stiffness of the bearing.

	Bearing 1	Bearing 2	Bearing 3	Bearing 4
Value /×10^7^ N·m^-1^	6.204	6.525	6.204	6.525

The mesh stiffness of the single side gear meshing pair with STP equal to zero is obtained from reference [30]. And the mesh stiffness of the GSTPS is calculated from section 2. The profile errors of the gears are obtained from reference [32].

### The steady vibration results with different STP

**Fig 11:**
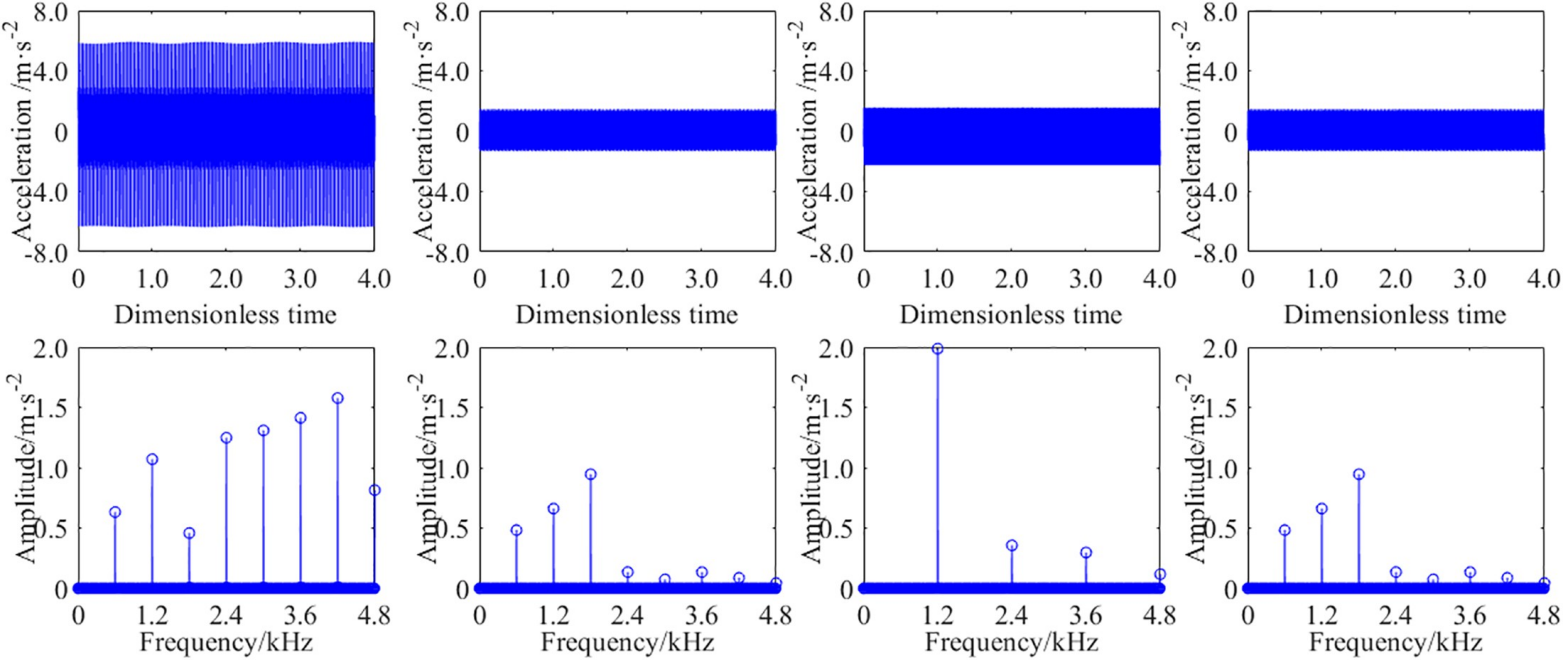
The analytical results of the vibration acceleration with different STP. (a) *p* = 0, (b) *p* = 0.25, (c) *p* = 0.50, (d) *p* = 0.75.

**Fig 12:**
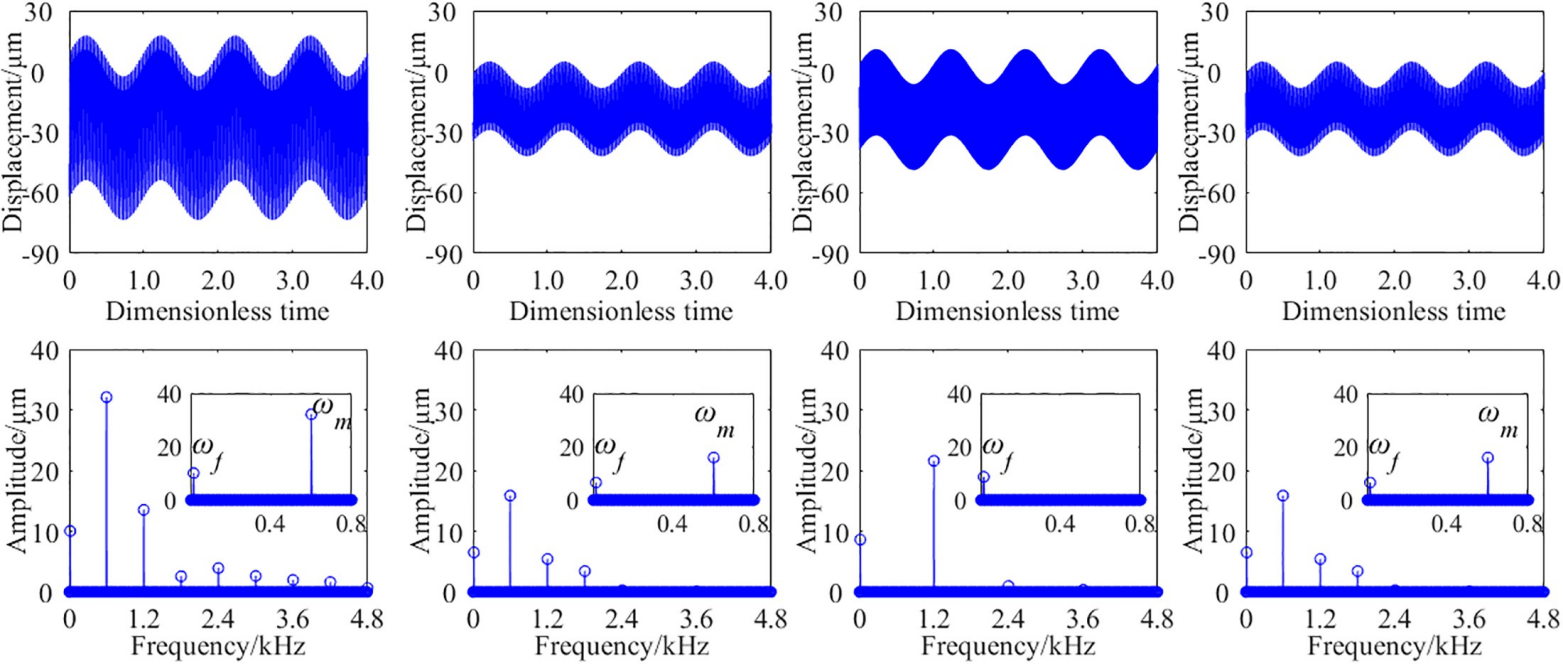
The analytical results of the vibration displacement with different STP. (a) *p* = 0, (b) *p* = 0.25, (c) *p* = 0.50, (d) *p* = 0.75.

In order to study the effect of the STP on the steady-state vibration response of a single stage spur gear pair, a certain working condition was selected, such as input speed of 500 rpm and a load of 20 Nm. The vibration response of the gear transmission system with the STP *p* = 0, *p* = 0.25, *p* = 0.50, and *p* = 0.75 are obtained from analytical method. Due to the fact that both the *x* and *y* directions are radial, the results obtained along both directions are similar. To avoid redundancy, the results of the vibration displacements and acceleration are only listed in one direction in the manuscript. Fig 11 shows the time-domain and frequency-domain results of the vibration acceleration at the output bearing node. Fig 12 shows the time-domain and frequency-domain results of the vibration displacement at the output bearing node.

When the STP are *p* = 0, *p* = 0.25, *p* = 0.50 and *p* = 0.75, respectively, the root mean square(RMS) of the vibration accelerations of the output bearing node are 1.93 m·s^-2^, 1.25 m·s^-2^, 1.55 m·s^-2^, 1.23 m·s^-2^, correspondingly. And the RMS of the vibration displacement are 22.89μm, 14.34μm, 16.99μm, 13.92μm, correspondingly. Compared with the results of the STP *p* = 0, the vibration acceleration RMS decreases by about 35.23%, 19.69%, 36.27% with *p* = 0.25, *p* = 0.50 and *p* = 0.75, and the vibration displacement RMS decreases by about 37.35%, 25.78%, and 39.19%, respectively. From the frequency domain diagram, those could be seen that shaft frequency and meshing frequency are the main frequencies that excite vibration. Obviously, for GSTPS, the amplitude of meshing frequency is significantly smaller than that of non-staggered structure, and some meshing frequency doubling is cancelled out.

### Comparison of vibration reduction trends with different STP

**Fig 13:**
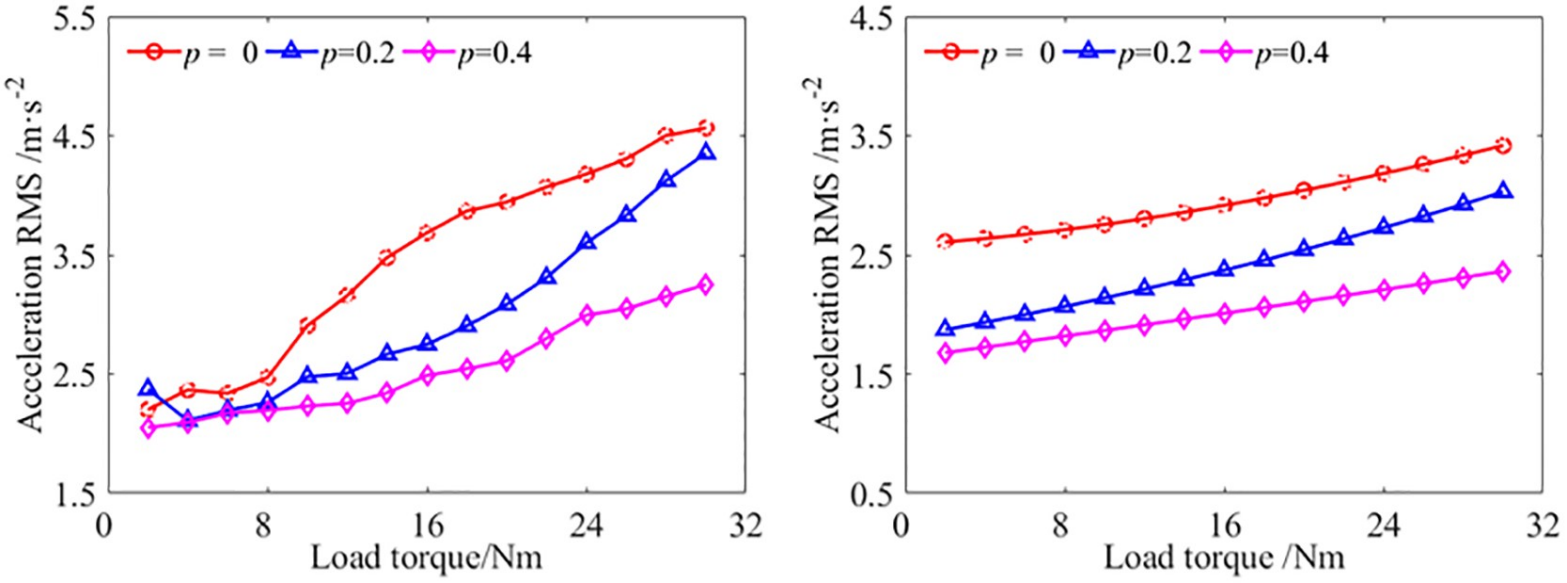
The vibration acceleration at the output bearing node with different load[S1]. (a) Experimental results, (b) Analytical results.

With the STP value *p* = 0, *p* = 0,2, and *p* = 0.4, respectively, the vibration acceleration at the output bearing node of the gear transmission system under different load are studied. The experiment results and analytical results are shown in Fig 13.

Comparing the vibration acceleration RMS at the output bearing node of the experimental results and analytical results under different load, it was found that regardless of the value of the STP, the vibration acceleration increases with the increase of the load. No matter the results from experiment or from the analytical simulation, the vibration acceleration RMS with the value of the STP *p* = 0.2 and *p* = 0.4 are lower than the result with *p* = 0, which verifies that the theoretical model of the GSTPS has good consistency with the experimental vibration reduction trend. Compared to the experimental results with *p* = 0, the experimental results with *p* = 0.2 and *p* = 0.4 have an average reduction of about 15.9% and 28.6%, respectively. Correspondingly, the analytical results have an average reduction of about 19.4% and 32.3%. the results indicate that the GSTPS could significantly reduce the vibration acceleration of spur gear pairs. However, the experimental results are greater than the simulation results due to installation errors, misalignment of the shaft, bearing clearance, and other issues during the processing and assembly of the experimental gear, which were not considered in the analytical model.

**Fig 14:**
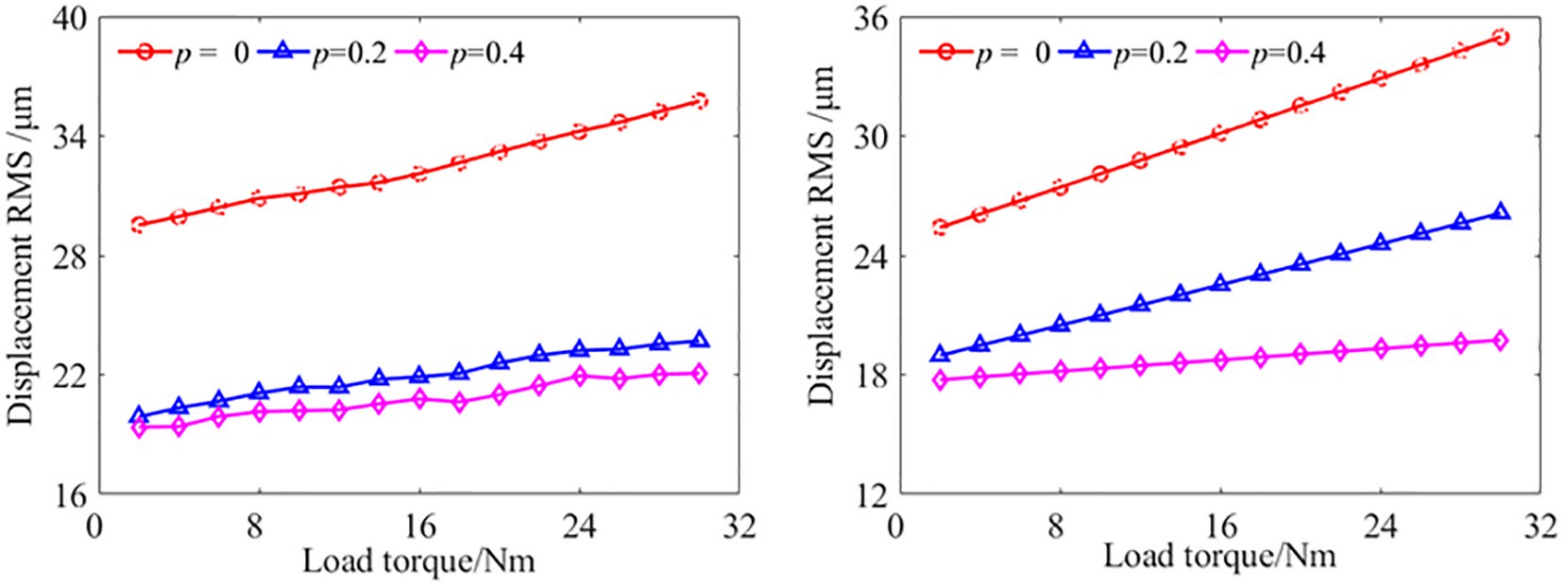
The vibration displacement at the output bearing node with different load[S1]. (a) Experimental results, (b) Analytical results.

With the STP value *p* = 0, *p* = 0,2, and *p* = 0.4, respectively, the vibration displacements at the output bearing node of the gear transmission system under different load are studied. The experiment results and analytical results are shown in Fig 14.

Comparing the vibration displacements RMS at the output bearing node of the experimental results and analytical results under different load, it was found that regardless of the value of the STP, the vibration displacements increases with the increase of the load. Both experimental and analytical results show that the vibration displacement with *p* = 0.2 and *p* = 0.4 are smaller than that with *p* = 0. This verifies that the theoretical model of the GSTPS has good consistency with the experiment on reducing vibration trend from the perspective of vibration displacement. Compared to the experimental results with *p* = 0, the experimental results with *p* = 0.2 and *p* = 0.4 decreased by an average of about 36.7% and 41.0%, respectively. The average reduction in analytical results are about 24.8% and 34.2%, respectively. The results indicate that the GSTPS can significantly reduce the vibration displacement of spur gear pairs.

**Fig 15:**
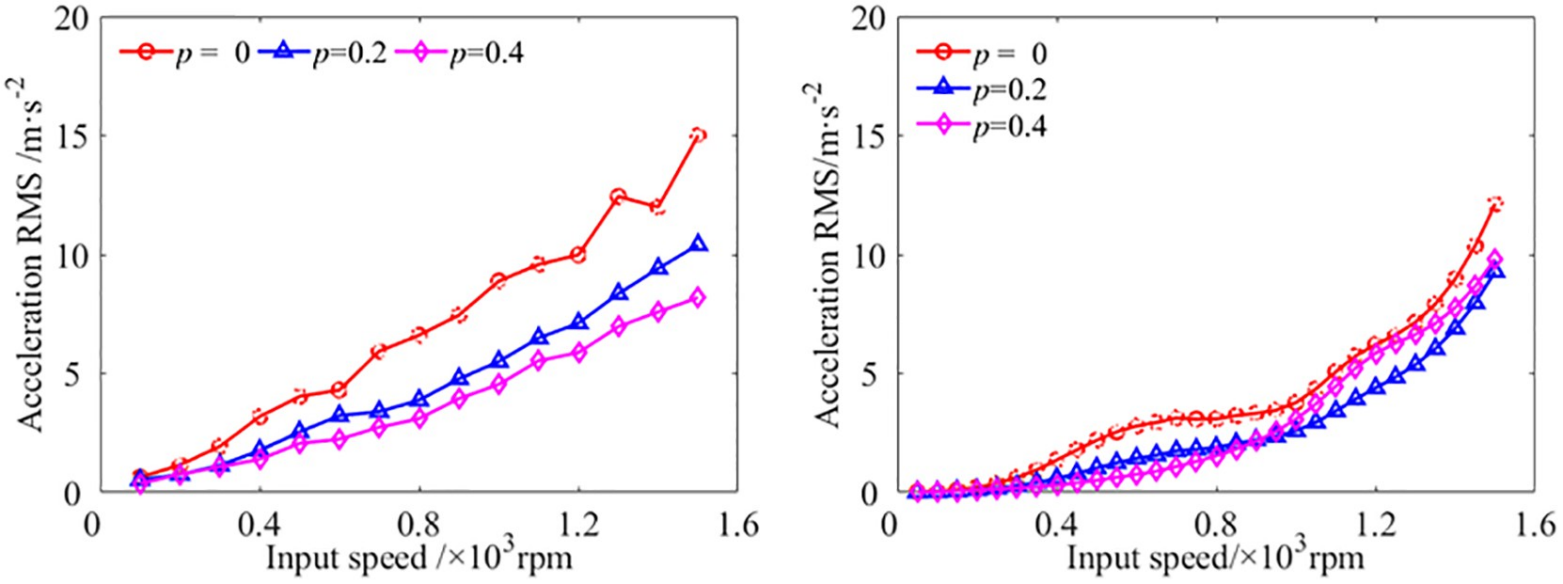
The vibration acceleration at the output bearing node with different input speed[S1]. (a) Experimental results, (b) Analytical results.

With the STP value *p* = 0, *p* = 0,2, and *p* = 0.4, respectively, the vibration accelerations at the output bearing node of the gear transmission system under different input speed are studied. The experiment results and analytical results are shown in Fig 15.

Comparing the experimental results and analytical results of vibration acceleration at different input speeds at the output bearing node, it could be found that regardless of the value of the STP, the vibration acceleration RMS increased with the increase of input speed. Regardless of the experimental and analytical results, the theoretical model of the GSTPS has good consistency with the experiment on reducing vibration trend. The vibration accelerations with *p* = 0.2 and *p* = 0.4 are lower than the result with *p* = 0, which verified that the GSTPS could significantly reduce the vibration acceleration of spur gear pairs.

### The law of the optimal STP

**Fig 16:**
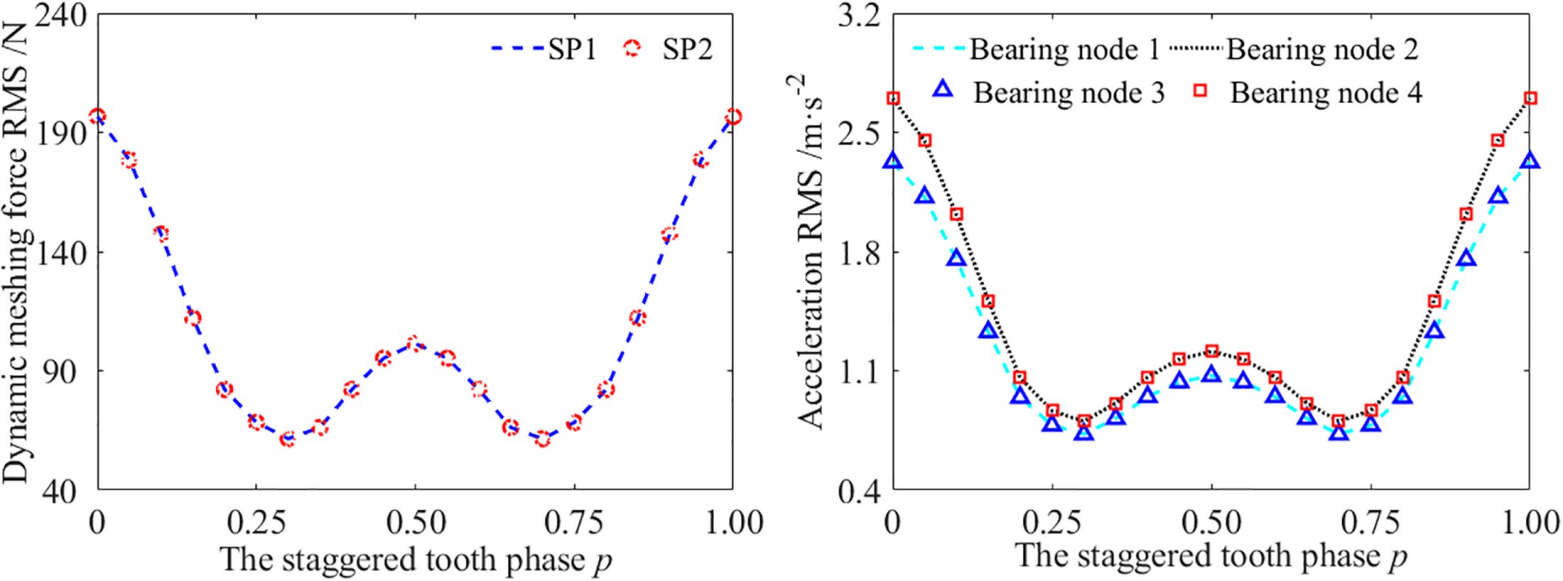
The dynamic response of the gear transmission system with different STP. (a) The dynamic meshing force, (b) The acceleration RMS.

To study the influence of the STP on the dynamic response of parallel axis spur gear pairs, the dynamic meshing force and vibration acceleration of the bearing nodes were extracted with different STP. It could be observed that the dynamic meshing force and the vibration acceleration of the bearing nodes do not always decrease with the increase of the STP, but show a trend of first decreasing and then increasing, with a turning point at *p* = 0.30. Moreover, the shape of symmetry is shown with *p* = 0.5 as the axis of symmetry, as shown in Fig 16.

**Fig 17:**
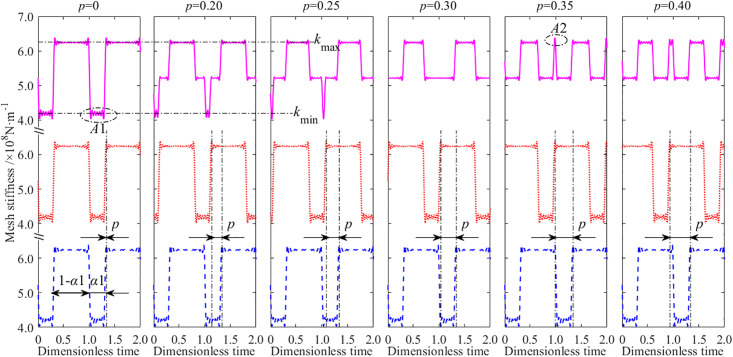
The mesh stiffness of the GSTPS with different STP. The top line (pink solid line) denotes the comprehensive mesh stiffness curve of the GSTPS. The middle line (Red dotted line) denotes the mesh stiffness of one meshing pair of the GSTPS. The bottom line (Blue dashed line) denotes the mesh stiffness of another meshing pair of the GSTPS.

Due to the fact that the time-varying mesh stiffness is the main factor causing changes in dynamic meshing force and vibration acceleration, the time domain diagrams of the mesh stiffness curves near *p* = 0.3 are given, as shown in Fig 17.

From the time domain diagram of the time-varying mesh stiffness, it could be found that when the STP equal to zeros, the time scope of the minimum value *k*_min_ of the mesh stiffness of the GSTPS is A1 = α_1_. This is consistent with the mesh stiffness curve of a single meshing pair. As the STP increases, the scope A1 of the minimum mesh stiffness *k*_min_ of the GSTPS gradually decreases, and the fluctuation of the dynamic meshing force and vibration acceleration decreases until the scope A1 disappears at *p* = 0.3. If the STP continues to increase, a scope A2 appears at the maximum value of the mesh stiffness *k*_max_. As the STP increases, the scope A2 increases, and the fluctuation of the dynamic meshing force and vibration acceleration also increases. Therefore, it is reasonable to infer that when the scope A1 disappears and the scope A2 appears, the corresponding STP minimizes the fluctuation of the dynamic meshing force and vibration acceleration. This STP is called the optimal STP of this meshing pair.

According to the variation law of the time-varying mesh stiffness curve of the GSTPS, it could be found that the moment when scope A1 of the minimum value disappears and the scope A2 of the maximum value appears, the STP corresponds to the optimal STP *p* = *α*_1_. Obviously, this only applies to *α*_1_<1-*α*_1_ (namely *α*_1_<0.5). if *α*_1_>1-*α*_1_ (namely *α*_1_>0.5), the optimal STP should be taken as *p* = 1-*α*_1_. The optimal STP of a spur gear pair is *p* = min(*α*_1_, 1-*α*_1_).

## Conclusion

A novel gear structure to reduce vibration is proposed, and the dynamic model of the GSTPS is established. The calculation formula of the time-varying mesh stiffness of the GSTPS is derived. The influences of the STP on the vibration response of a single gear pair are studied. And the test bench of the GSTPS is built to verify the correctness of the theoretical model. The main conclusions obtained are as follows:

1. The time-varying mesh stiffness of the GSTPS is not only related to the contact ratio, but also to the value of the STP.
2. The GSTPS could changes the frequency of the vibration response of the transmission system. The odd multiple frequency of the meshing frequency disappears, and the amplitude of the even multiple frequency of the meshing frequency increases with STP *p* = 0.5.
3. The GSTPS has an effect to reduce vibration with different input speed and load. And the higher the input speed and load torque, the more obvious effect on reducing vibration.
4. The optimal STP of a stage spur gear pair is obtained, which is *p* = min(α1, 1-α1).

The results of the study have a certain guiding significance in the structural design of gear transmission systems and gear vibration reduction.

## Supporting information

S1 Data (XLSX)
